# PKCε Promotes HuD-Mediated Neprilysin mRNA Stability and Enhances Neprilysin-Induced Aβ Degradation in Brain Neurons

**DOI:** 10.1371/journal.pone.0097756

**Published:** 2014-05-21

**Authors:** Chol Seung Lim, Daniel L. Alkon

**Affiliations:** Blanchette Rockefeller Neurosciences Institute at West Virginia University, Morgantown, West Virginia, United States of America; Boston University School of Medicine, United States of America

## Abstract

Amyloid-beta (Aβ) peptide accumulation in the brain is a pathological hallmark of all forms of Alzheimer’s disease. An imbalance between Aβ production and clearance from the brain may contribute to accumulation of neurotoxic Aβ and subsequent synaptic loss, which is the strongest correlate of the extent of memory loss in AD. The activity of neprilysin (NEP), a potent Aβ-degrading enzyme, is decreased in the AD brain. Expression of HuD, an mRNA-binding protein important for synaptogenesis and neuronal plasticity, is also decreased in the AD brain. HuD is regulated by protein kinase Cε (PKCε), and we previously demonstrated that PKCε activation decreases Aβ levels. We hypothesized that PKCε acts through HuD to stabilize NEP mRNA, modulate its localization, and support NEP activity. Conversely, loss of PKCε-activated HuD in AD leads to decreased NEP activity and accumulation of Aβ. Here we show that HuD is associated with NEP mRNA in cultures of human SK-N-SH cells. Treatment with bryostatin, a PKCε-selective activator, enhanced NEP association with HuD and increased NEP mRNA stability. Activation of PKCε also increased NEP protein levels, increased NEP phosphorylation, and induced cell surface expression. In addition, specific PKCε activation directly stimulated NEP activity, leading to degradation of a monomeric form of Aβ peptide and decreased Aβ neuronal toxicity, as measured by cell viability. Bryostatin treatment also rescued Aβ-mediated inhibition of HuD-NEP mRNA binding, NEP protein expression, and NEP cell membrane translocation. These results suggest that PKCε activation reduces Aβ by up-regulating, via the mRNA-binding protein HuD, Aβ-degrading enzymes such as NEP. Thus, PKCε activation may have therapeutic efficacy for AD by reducing neurotoxic Aβ accumulation as well as having direct anti-apoptotic and synaptogenic effects.

## Introduction

Alzheimer's disease (AD) is a progressive neurodegenerative disease characterized by insidious cognitive decline and memory dysfunction [1]. Synapse loss is the best pathological correlate of cognitive decline in AD and mounting evidence suggests that AD is primarily a disease of synaptic dysfunction [2]–[4]. Soluble oligomeric forms of amyloid-beta (Aβ) peptide, the peptide that aggregates to form senile plaques in the brain of AD patients, have been shown to be toxic to neuronal synapses both *in vitro* and *in vivo* and accumulation of Aβ peptide plays a central role in the development of the disease [5]–[7]. Therefore, preventing the accumulation of the monomeric form of Aβ peptide in the brain has the potential to prevent neuronal death and memory loss in AD [8].

Several enzymes degrade Aβ peptide, including angiotensin-converting enzyme (ACE), endothelin-converting enzyme (ECE), insulin-degrading enzyme (IDE), matrix metalloproteinases (MMPs), neprilysin (NEP), and plasmin [5], [9]–[11]. Of these, NEP has been reported as the major physiological Aβ peptide-degrading enzyme in the brain [12], [13]. Previous studies have shown that this enzyme is down-regulated in areas vulnerable to Aβ peptide accumulation in the AD brain [14]–[17]. Thus, increasing the expression and activity of NEP in the AD brain may prevent the accumulation of Aβ peptide, protect neurons against Aβ toxicity, and help reverse Aβ-related synaptic loss and cognitive deficits.

We have shown that chronic treatment of Tg2576 AD mice and an aged rat model with bryostatin, a selective PKCε activator, dramatically reduces the levels of Aβ, recovers the loss of neurotrophic activity and synapses, and enhances cognitive function [18], [19]. We also previously demonstrated that PKCε activation up-regulates HuD expression and stimulates its redistribution into the cytosol and dendrites of hippocampal neurons, where it subsequently controls post-transcriptional expression of target genes [20]. HuD is an mRNA binding protein that plays a pivotal role in processing, transport, stability, and local translation of various mRNAs important for synaptogenesis and neuronal plasticity in the brain [21]–[23], such as BDNF and NGF [24] that are important for neuronal survival, growth and differentiation, and normal neuronal development [25]–[27]. The expression levels of HuD in the brain decrease with age and in the early stages of AD [12], [28]. We hypothesized that PKCε-induced stabilization of NEP mRNA via HuD protein increases active NEP protein and activity, thereby abrogating the neurotoxic effects of Aβ. In the AD brain, elevated Aβ peptide directly binds to a putative protein kinase C (PKCε) substrate domain (Aβ 28–30) and inhibits PKCε translocation and activation [29]. Decreased PKCε leads to a loss of HuD activation; thus, we further hypothesized that as Aβ accumulates in AD, the subsequent loss of PKCε and HuD activity leads to a loss of NEP mRNA stability and activity that permits further Aβ accumulation and disease progression. We utilized cultures of human neuroblastoma cells to determine the mechanistic relationship between PKCε, HuD, and NEP and their roles in Aβ degradation.

## Materials and Methods

### Cell Culture and Treatments

Human neuroblastoma SK-N-SH cells were obtained from American Type Culture Collection (ATCC, HTB-11) and were grown in minimum essential medium supplemented with 1 µM non-essential amino acids, 100 UI/mL penicillin, 100 µg/mL streptomycin, and 10% fetal bovine serum (all culture materials from Invitrogen) under a humidified atmosphere of 5% CO_2_/95% air at 37°C. Cells were subcultured 2 times per week and only cells between passages 4 and 8 were used in experiments. Cells were treated with combinations of bryostatin (Enzo Life Sciences), actinomycin D (Enzo Life Sciences), Ro 32–0432 (Bisindolylmaleimide XI. hydrochloride, Enzo Life Sciences), monomeric or oligomeric Aβ (AnaSpec), or phosphoramidon (Sigma-Aldrich).

### Preparation of Aβ

For each experiment, monomeric and oligomeric Aβ were prepared from aliquots of the same batch of Aβ. To prepare oligomeric Aβ, lyophilized Aβ aliquots (0.3 mg) were dissolved in 0.2 mL of 1,1,1,3,3,3-Hexafluoro-2-propanol (HFP, Sigma-Aldrich) and then added to 0.7 mL of H_2_O. Samples were loosely capped and stirred on a magnetic stirrer under a fume hood for 48 hr and then used within 36 hr. Monomeric Aβ was prepared immediately before use by rapidly evaporating the HFP via gently bubbling nitrogen gas into the solution. The quality of Aβ preparations were routinely checked by dot-blot and immunoblot with A-11 (1∶1,000, Invitrogen) and 6E10 (1∶1,000, Covance) antibodies. The final concentration of the oligomeric Aβ preparation was nominally calculated based on the concentration of the starting Aβ monomer.

### Real-time Quantitative Reverse Transcription-polymerase Chain Reaction (RT-qPCR)

Total RNA was isolated using the RNeasy mini kit (Qiagen) per the manufacturer’s protocol. For RT reactions, 500 ng of total RNA was reverse transcribed using oligo(dT) primer and Superscript III (Invitrogen) at 50°C for 1 hr. Real-time PCR was performed for 40 cycles with SYBR Green 1 PCR master mixture and processed on LightCycler 480 II (Roche) machine using specific primers against human NEP, GAPDH, or histone (all from Qiagen). Reactions were run in triplicate for each sample and a dissociation curve was generated. Threshold cycles (Ct) for NEP amplification were normalized on the house keeping GAPDH (dCt) and every experimental sample was referred to its control (ddCt). Relative expression change values were expressed as 2^−ddCt^.

### RNA Binding Protein Immunoprecipitation (RIP) Assay

The RIP assay was performed as described previously [24]. In brief, cultured cells were lysed in cold lysis buffer (50 mM Tris-HCl, pH 8.0, 150 mM NaCl, 100 mM sodium fluoride, 1 mM sodium orthovanadate, 1 mM EGTA, 1 mM EDTA, 1% Triton X-100, 2 mM phenylmethylsulfonyl fluoride, 10 mM vanadyl ribonucleoside complex, 1X complete protease inhibitor cocktail, and 1X RNase inhibitor [Sigma-Aldrich]) for 15 min at 4°C. Lysates were spun at 14,000×*g* for 15 min at 4°C, and supernatants were incubated overnight with rabbit anti-HuD polyclonal antibody (Santa Cruz) at 4°C with gentle rotation. After washing, HuD protein/mRNA complexes were purified using protein A-magnetic beads and a magnetic separator (both from Millipore). Total RNA was extracted from the immunocomplexes with Trizol (Sigma-Aldrich) following the manufacturer’s instructions and then used for RT-qPCR as described above.

### RNA Electrophoretic Mobility Shift Assays (RNA-EMSA)

Oligoribonucleotides containing the candidate adenine- and uridine-rich instability-conferring element sequence (AU-rich element; ARE) from human NEP (5'- UUCUGUGAUCAUUUAUUUUAAGCACUC-3'; NM_000902, 2933–2959) were synthesized and labeled with biotin at the 3′-end (Sigma-Aldrich). As a negative control without the ARE sequence, human GAPDH 3′-UTR (NM_002046) construct (220 bp, Origene) was used for biotin-labeled ribonucleotide synthesis using the MEGAshortscript kit (Life Technologies) following the manufacturer’s instructions. Biotin-labeled RNA probe (125 nM) was incubated with purified HuD protein (2 µM, Origene) in binding buffer (10 mM HEPES, pH 7.3; 20 mM KCl, 1 mM MgCl2, 1 mM DTT, 5% glycerol, 2 µg/ml tRNA) for 30 min at room temperature. Unlabeled RNA probe was used as a competitor in each reaction. After incubation, the RNA-protein mixture was electrophoresed in a 6% native polyacrylamide gel (Invitrogen) in 0.5X TBE and transferred to a positively charged nylon membrane. Signals on the blots from the transferred membrane were detected using a chemiluminescence nucleic acid detection module (Thermo Scientific) according to the manufacturer’s protocol.

### HuD, PKCε, NEP Gene Silencing

Human HuD-, PKCε-, and NEP-specific siRNA and scrambled control siRNA were purchased from Santa Cruz. The gene silencing conditions for each gene were determined by electroporation using the Nucleofector system (Lonza) according to manufacturer's specifications. Briefly, SK-N-SH cells (1×10^5^ cells/ml) were harvested and resuspended in 100 µl of Nucleofector transfection solution. After transfection of HuD, PKCε, NEP, or scrambled control siRNA (20 nM), cells were immediately plated in dishes containing complete media. The following day cells were split, and 72 hr later either lysed or subjected to further analyses, as described.

### mRNA Stability Assay

mRNA stability was measured by RT-qPCR after the addition of the transcriptional inhibitor actinomycin D (10 µg/ml) with/without bryostatin (0.5 nM) to cultured cells for 2, 4, 6, 8, or 10 hr. To see the effect of PKCε activity, some cells were pre-treated with a PKCε inhibitor Ro 32–0432 (2 µM) for 2 hr and further analyzed by RT-qPCR. The amount of NEP mRNA at each time point was compared with the initial mRNA level (100%). A nonlinear regression analysis was conducted, which gave a first-order mRNA decay constant (*k*). Average mRNA half-life (t_1/2_) was calculated as 0.693/*k* from the equation Mt = M_0_×e^−*k*t^.

### Membrane Protein Biotinylation and Detection

Cells treated as above were washed three times with ice-cold PBS and incubated with PBS containing 1 mg/ml EZ-Link sulfo-N-hydroxysuccinimide-SS-biotin (Thermo Scientific) at 4°C for 90 min, according to the manufacturer’s instructions. Cells were then washed three times with ice-cold PBS supplemented with 50 mM Tris (pH 8.0) and finally lysed on ice with lysis buffer for immunoprecipitation of NEP.

### Co-immunoprecipitation (IP) and Immunoblot Analysis

Cultured cells were lysed in cold lysis buffer (10 mM Tris-HCl, pH 7.4, 5 mM EDTA, 1% Triton X-100, 10% glycerol, 1 mM CaCl_2_, 1 mM MgCl_2_, 1X complete protease inhibitor cocktail, and 1X phosphatase inhibitor cocktail [Thermo Scientific]) for 1 hr at 4°C. Total lysates (1 mg) were immunoprecipitated with anti-NEP (Abcam) antibody, and protein/antibody immunocomplexes were purified with protein A-magnetic beads and a magnetic separator (both from Millipore). After washing, immunocomplexes were separated by 10% sodium dodecyl sulfate-polyacrylamide gel electrophoresis (SDS-PAGE), transferred onto nitrocellulose membranes, and incubated with mouse monoclonal anti-NEP (1∶1,000, Millipore), mouse monoclonal anti- Aβ (6E10, 1∶1,000, Covance), or rabbit phospho-Ser/Thr (1∶500, Abcam) antibodies. After incubation with horseradish peroxidase (HRP)-conjugated goat anti-rabbit or mouse IgG (1∶10,000; Jackson ImmunoResearch Lab), the membranes were developed with Enhanced Chemiluminescence (ECL) Substrate and exposed to X-ray film (Thermo Scientific). To detect biotinylated NEP proteins, the membranes were directly incubated with streptavidin-HRP (Invitrogen), developed with ECL Substrate, and exposed to X-ray film. As a control, membranes were stripped and re-probed with mouse anti-NEP or mouse anti-α-tubulin (1∶5,000, Sigma-Aldrich) antibodies followed by HRP-conjugated goat anti-mouse IgG (1∶10,000; Jackson ImmunoResearch Lab). Levels of immunoreactivity were assessed by densitometric analysis of films using an HP Scanjet densitometer and Image-J image analysis system software (1.44a, NIH).

### NEP Activity Assay

NEP activity was measured as described previously [30]. Briefly, after treatment with PKCε activator bryostatin with/without Ro 32–0432 or phosphoramidon for the indicated times, intact cells were washed and incubated with 1 mM glutaryl-Ala-Ala-Phe-4-methoxy-2-naphthylamide (Sigma-Aldrich) solution as a NEP substrate. The substrate solution was collected and incubated with leucine aminopeptidase (50 µg/ml, Sigma-Aldrich) in the absence or presence of 10 µM phosphoramidon for 30 min at 37°C and the released free 4-methoxy-2-naphthylamide was measured fluorometrically at an emission wavelength of 425 nm using a microplate reader (BioTek).

### Aβ Peptide Degradation Assay

Cells were incubated with 2.5 µg of the monomeric form Aβ peptide 1–42 in a final volume of 100 µl of 50 mM HEPES buffer (pH 7.2) at 37°C for 4 hr. The reactions were carried out in the presence or absence of phosphoramidon (10 µM). Proteins and peptides in the reaction were precipitated using an equal volume of 20% trichloroacetic acid (TCA, Sigma-Aldrich). All pellets were resuspended in 2X SDS protein-loading buffer followed by immunoblot analyses using anti-Aβ peptide antibody 6E10 (1∶1,000, Covance) as described above.

### Cell Toxicity Assay

Cells were cultured in 48- or 96-well plates at a density of ∼3,000–5,000 cells/well in complete growth medium for 24 hr. Then, growth media was replaced with fresh culture media (∼100–200 µl/well) containing 2.0% FBS and 1 µM oligomeric Aβ peptides 1–42. After 24 hr, cell viability assay was performed as in a previous study [31] using Cell Counting Kit-8 (Dojindo) by adding ∼10–20 µl of CCK-8 solution to all wells. The plates were then incubated for 4 hr at 37°C in 5% CO_2_ and culture medium was collected and used to measure the absorbance at 450 nm on a microplate reader (BioTek). The difference in OD relative to untreated controls was taken as a measure of cell viability and the percentage of cell viability was calculated by comparing the ODs at 450 nm of the wells treated with Aβ peptides with those of controls.

### Quantification and Statistical Analysis

Quantitative data are expressed in arbitrary units (%) comparing untreated controls with cells treated with bryostatin or the indicated concentrations of inhibitors. All data are presented as mean ± SEM from 3 or more independent experiments unless otherwise indicated. Statistical comparisons between different treatment groups were conducted with Tukey's multiple comparison tests after one-way ANOVA using GraphPad Prism 5 software (GraphPad Software Inc.). P values of less than 0.05 were considered to be statistically significant.

## Results

### HuD Protein is Specifically Associated with NEP mRNA

**Figure 1:**
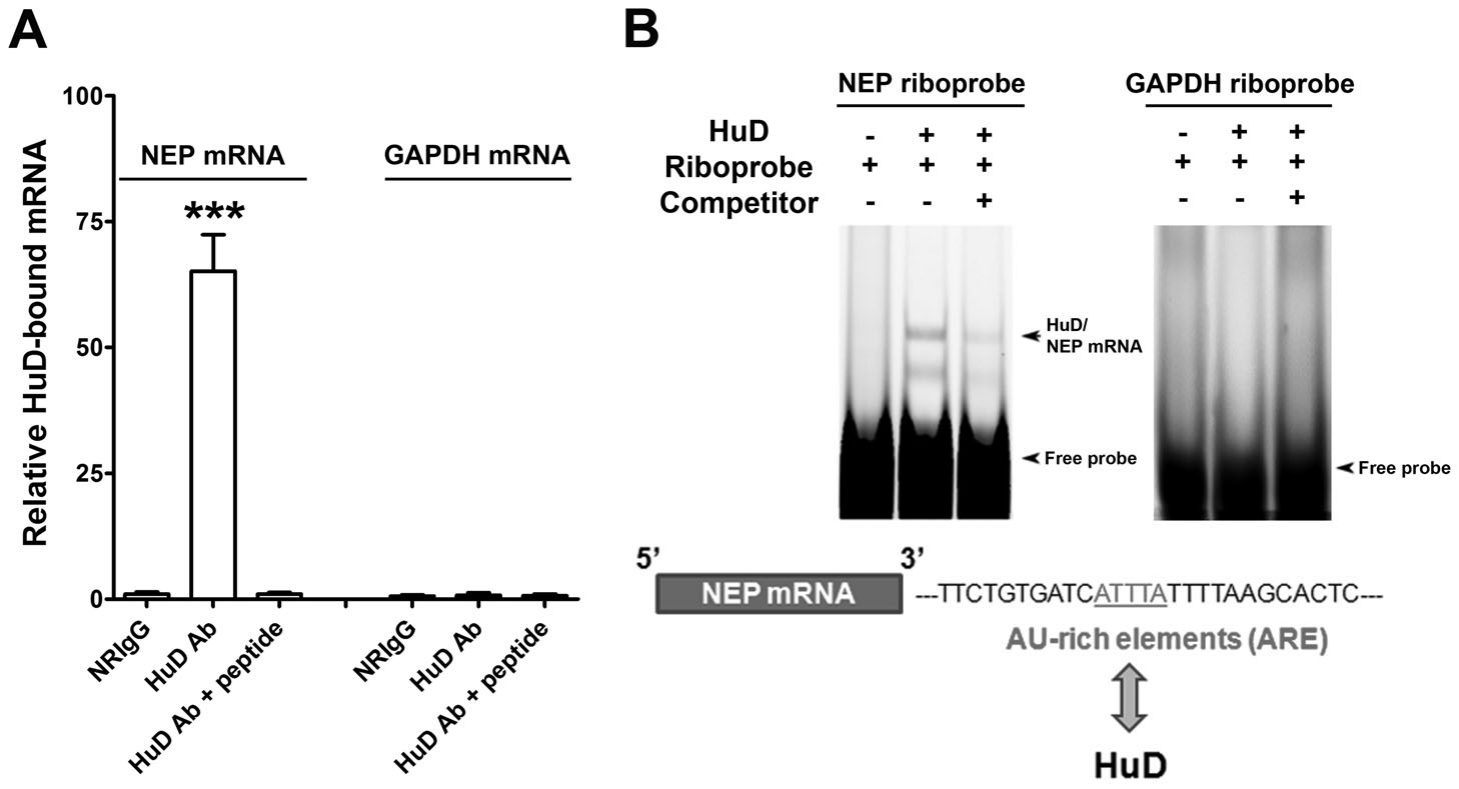
HuD protein specifically binds to NEP mRNA in human SK-N-SH cells. **A**, RIP assay showing HuD-bound NEP mRNA in cultured SK-N-SH cells. Cells were lysed and immunoprecipitated with normal rabbit immunoglobulin G (NRIgG), HuD antibody, or antibody solution with HuD peptide and then total RNA was isolated from immunoprecipitates and used for RT-qPCR with specific human NEP primers. As a control, human GAPDH mRNA was amplified from RT-qPCR of total RNA (Mean ± SEM of three independent experiments, ***P<0.001, compared with NRIgG). **B**, RNA-EMSA assay using recombinant HuD protein and a biotin-labeled oligoriboprobe corresponding to the candidate ARE sequence in 3′-UTR region of NEP mRNA and the 3′-UTR of GAPDH mRNA as a negative control, respectively. Unlabeled oligoriboprobe was used as a competitor for the inhibition reaction.

To determine whether NEP expression is regulated in human neuroblastoma cells at the mRNA level by binding to HuD, we first conducted a RIP assay using a normal HuD antibody. HuD protein specifically interacted with NEP mRNA, but not with GAPDH mRNA (Fig. 1A). This interaction was confirmed using HuD antibody pre-absorbed with a sequence-specific inhibitory peptide.

HuD is known to bind AU-rich elements (ARE) sequence in the 3′-UTR of target mRNAs [32]. We synthesized a biotin-labeled oligoribonucleotide that includes the highly conserved sequence (AUUUA) ARE from the human NEP mRNA 3′-UTR and performed an RNA-EMSA assay using recombinant HuD protein. As a negative control, we used a biotin-labeled oligoribonucleotide for human GAPDH mRNA 3′-UTR, which does not contain an ARE sequence (Fig. 1B). HuD protein formed complexes with the NEP mRNA ARE sequence, but not with the GAPDH riboprobe. Specificity of the HuD-mRNA interaction was confirmed with the addition of an unlabeled competitor oligoriboprobe.

### PKCε Activation Stabilizes NEP mRNA through HuD Activation

**Figure 2:**
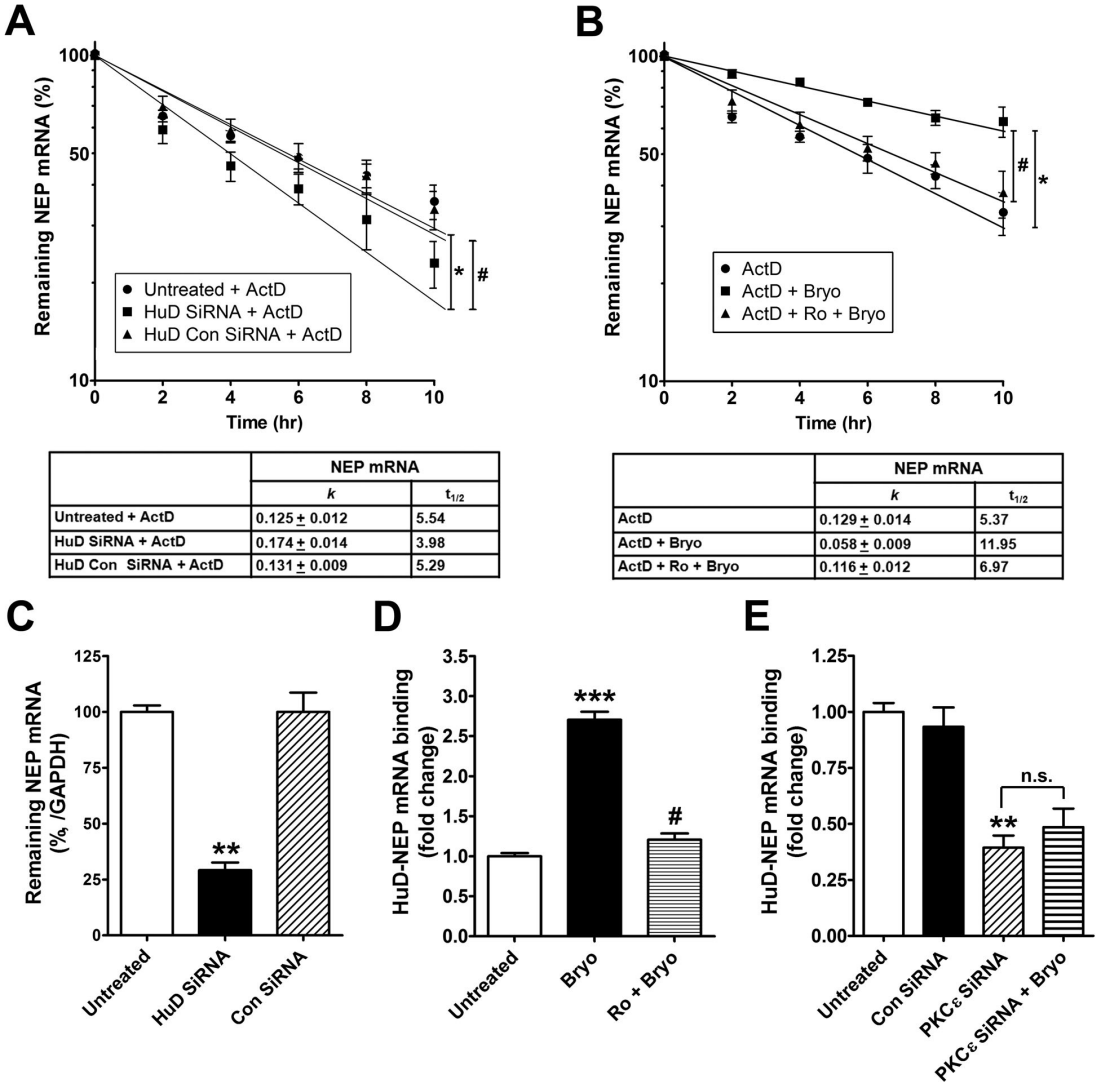
Figure 2. Activated PKCε stabilizes NEP mRNA. **A**, Cells were untreated or incubated with HuD siRNA or control siRNA (Con siRNA) for 4 days and then treated with actinomycin D (ActD, 10 µg/ml) for 2, 4, 6, 8, and 10 hrs. Total RNA was isolated and NEP mRNA was quantified by real time RT-qPCR. NEP mRNA at each time point was compared with the initial mRNA level (100%). A nonlinear regression analysis was conducted to calculate the first-order decay constant (*k*). Average mRNA half-life (t_1/2_) was calculated as 0.693/*k* and reported in the table (Mean ± SEM of the three independent experiments, *P<0.05, HuD siRNA+ActD compared with untreated; #P<0.05, HuD siRNA+ActD compared with Con siRNA-treated). **B**, mRNA stability assay from cells treated with ActD or ActD+bryostatin (Bryo, 0.5 nM), or pre-treated with Ro 32-0432 (Ro, 2 µM) for 30 min and then treated with ActD+Bryo for 2, 4, 6, 8, and 10 hrs. Average mRNA half-life (t_1/2_) was calculated as 0.693/*k* and reported in the table (Mean ± SEM of the three independent experiments, **P<0.01, ActD+Bryo compared with ActD to assess the bryostatin effect; ^#^P<0.01, ActD+Bryo compared with ActD+Ro+Bryo to assess the Ro 32-0432 effect). **C**, Quantitative RT-qPCR analysis showing remaining NEP mRNA from untreated cells or cells incubated with HuD siRNA or Con siRNA for 4 days (Mean ± SEM for three independent experiments, **P<0.01, compared with untreated). **D**, RIP analysis to detect NEP mRNA associated with HuD protein in untreated cells or cells treated with 0.5 nM Bryo or pre-treated with 2 µM Ro for 30 min and then treated with Bryo for 1 hr. Relative amounts of NEP mRNA bound to HuD were analyzed by real-time RT-qPCR (Mean ± SEM for three independent experiments, ***P<0.001, compared with untreated; ^#^P<0.001, compared with Bryo-treated). **E**, RIP analysis to detect NEP mRNA associated with HuD protein in untreated cells or cells incubated with PKCε siRNA or Con siRNA, and then treated without/with Bryo for 1 hr (Mean ± SEM for three independent experiments, **P<0.01, compared with untreated; n.s. = not specific).

To determine whether HuD protein is important for NEP mRNA stability and expression in SK-N-SH neuroblastoma cells, we suppressed HuD expression with specific siRNA against HuD, and then cultured the cells under transcriptional arrest (induced by actinomycin D). The decay of NEP mRNA during a 10-hr time course was measured in the cell lysates by RT-qPCR. As expected, actinomycin D treatment increased mRNA degradation kinetics, with a faster decrease in NEP mRNA half-life in cells transduced with HuD siRNA, compared with untreated or cells transduced with a scrambled control siRNA (Fig. 2A). We also performed RT-qPCR to determine the effect of HuD silencing on NEP mRNA levels. The cells transduced with HuD siRNA showed an approximate 75% decrease in NEP mRNA, compared with untreated cells or cells transduced with a scrambled control siRNA (Fig. 2C). These results indicate that HuD protein is involved in stabilization of NEP mRNA.

To verify whether PKCε is required for HuD-mediated NEP mRNA stability, cultured cells under transcriptional arrest (induced by actinomycin D) were treated without or with the PKCε activator bryostatin. The decay of NEP mRNA during a 10-hr time course was measured in the cell lysates by RT-qPCR. Actinomycin D treatment increased mRNA degradation kinetics, with a decrease in NEP mRNA half-life (Fig. 2B). When cells were co-treated with bryostatin, the decay rate of the NEP transcript was delayed, with a dramatic increase in mRNA half-life. The effect of bryostatin was completely inhibited by pre-treatment of cells with the PKCε inhibitor Ro 32-0432. This result suggests that PKCε activation is involved in stabilization of NEP mRNA.

To investigate whether PKCε stabilizes NEP mRNA by enhancing HuD binding, a RIP assay was performed with anti-HuD antibody using total mRNA from human cells treated with bryostatin for 1 hr. Bryostatin treatment induced HuD binding to NEP mRNA (approximately 3 fold; Fig. 2D). This effect was almost completely blocked in cells pre-treated with Ro 32-0432 for 30 min. We also tested this effect in cells transduced with PKCε siRNA compared to untreated cells or cells transduced with a scrambled control siRNA (Fig. 2E). Cells transduced with PKCε siRNA showed a 70% decrease in HuD-NEP mRNA binding that could not be rescued with bryostatin treatment.

**Figure 3:**
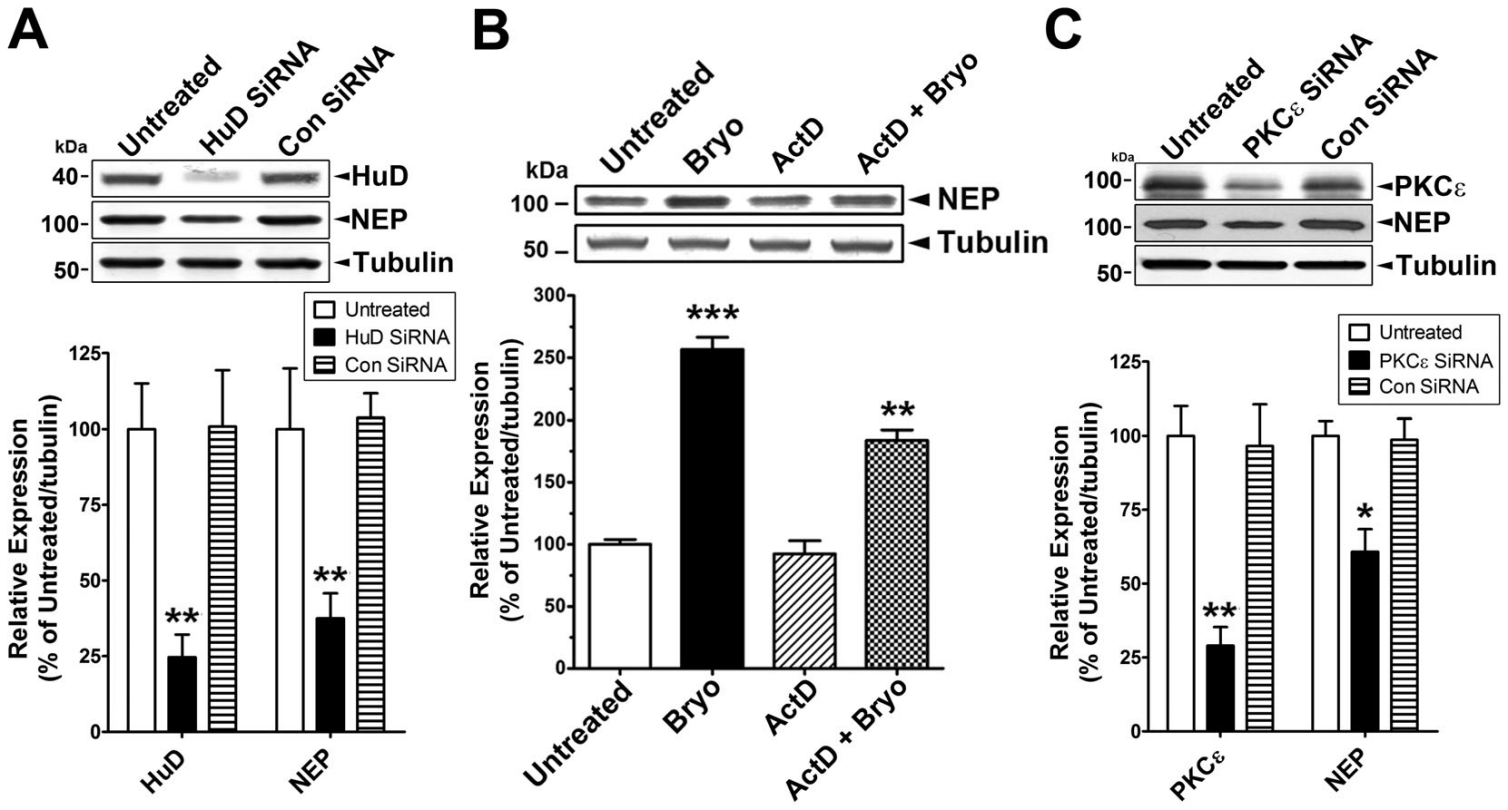
Figure 3. Activated PKCε enhances HuD binding to NEP mRNA and increases NEP protein expression. **A**, Immunoblot analyses to detect HuD and NEP protein levels in untreated cells or cells incubated with control siRNA (Con siRNA) or HuD siRNA (Mean ± SEM, **P<0.01, compared with untreated). **B**, Relative expression of NEP protein was measured by immunoblot analysis after treatment with bryostatin (Bryo, 0.5 nM) or actinomycin D (ActD, 10 µg/ml), or pre-treatment with ActD for 1 hr prior to bryostatin (ActD+Bryo) for 24 hr (Mean ± SEM of three independent experiments, **P<0.01, ***P<0.001, compared with untreated control). **C**, Immunoblot analyses to detect PKCε and NEP protein levels in untreated cells or cells incubated with Con siRNA or PKCε siRNA (Mean ± SEM, *P<0.05, **P<0.01, compared with untreated).

To further investigate whether HuD reduction affects NEP protein expression, we suppressed HuD expression with a specific siRNA against HuD and measured HuD and NEP protein levels by immunoblot analysis (Fig. 3A). HuD silencing decreased the protein levels of HuD and NEP by ∼80 and 70%, respectively. Treatment with bryostatin also led to a significant increase in NEP protein expression (Fig. 3B). To determine whether PKCε-stimulated binding of HuD to NEP mRNA affects post-transcriptional regulation, we measured NEP protein levels in cells transcriptionally arrested by pre-treatment with the transcriptional inhibitor actinomycin D for 1 hr prior to bryostatin treatment. Even when transcription is blocked, there was a significant increase in NEP protein level after bryostatin treatment. We also examined whether PKCε plays a role in NEP expression, measuring PKCε and NEP protein levels by immunoblot analysis under PKCε silencing conditions (Fig. 3C). Cells transduced with PKCε siRNA showed a decrease in NEP protein level of approximately 40%. Taken together, these results indicate that PKCε is involved in HuD-mediated NEP expression and the increased expression of NEP protein after PKCε activation is related to post-transcriptional regulation of existing NEP transcripts.

### Activated PKCε Increases Phosphorylation and Cell Membrane Localization of NEP

**Figure 4:**
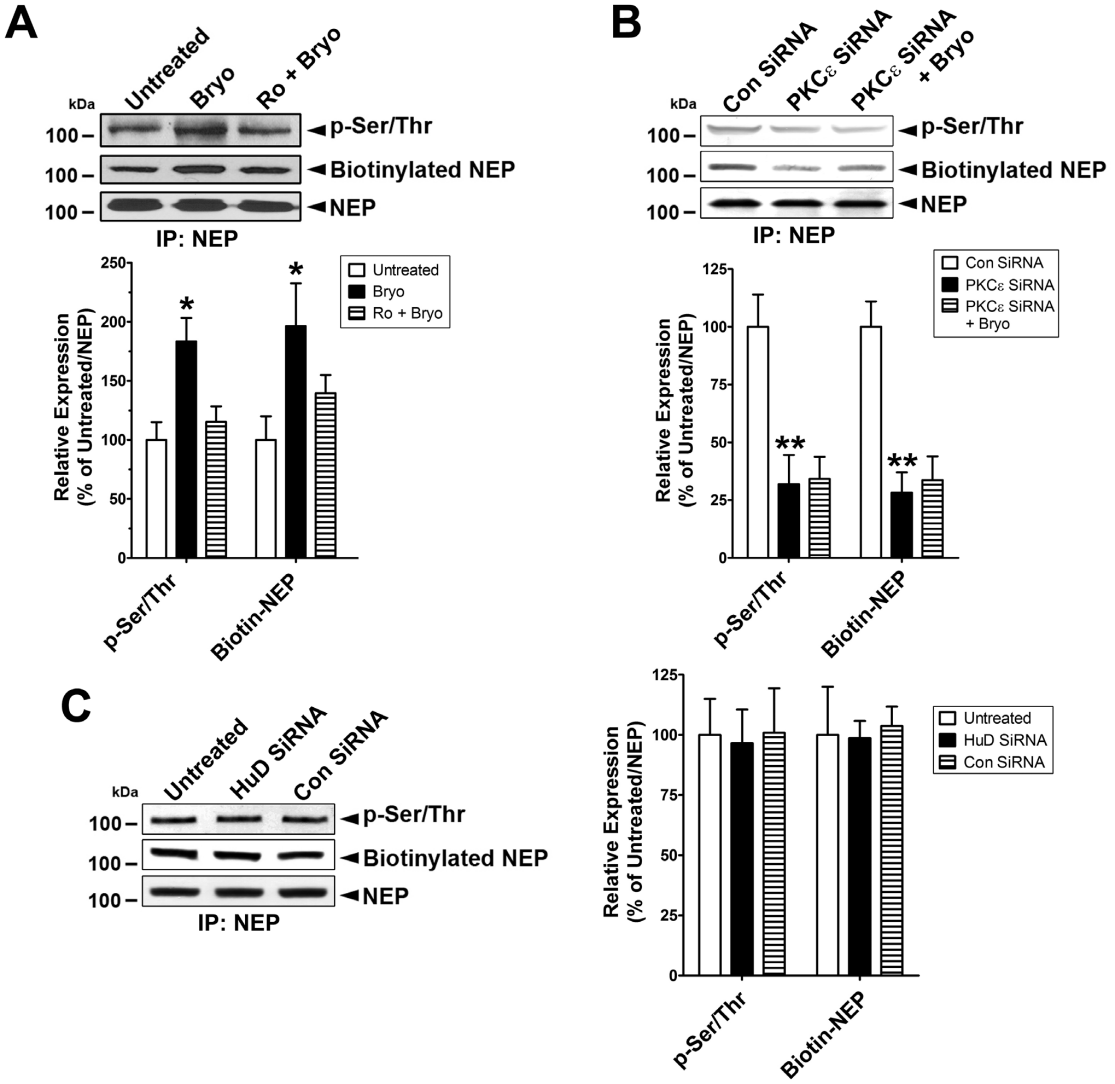
Activated PKCε increases phosphorylation and membrane localization of NEP. **A**, Cells were untreated or treated with bryostatin (Bryo, 0.5 nM) or pre-treated with Ro 32-0432 (Ro, 2 µM) for 30 min and then treated with Bryo for 1 hr, lysed, and then used for immunoprecipitation using NEP antibody. Immunocomplexes were further analyzed to detect phosphorylated and biotinylated NEP protein in immunoblot analyses after normalization for NEP protein level (Mean ± SEM, *P<0.05, compared with untreated). **B**, Cells were untreated or incubated with control siRNA (Con siRNA) or PKCε siRNA for 4 days without/with bryostatin (1 nM) treatment for 1 hr, lysed, and then used for immunoprecipitation using NEP antibody. Immunocomplexes were further analyzed to detect phosphorylated and biotinylated NEP protein in immunoblot analyses after normalization for NEP protein level (Mean ± SEM, **P<0.01, compared with Con siRNA). **C**, Cells were untreated or incubated with Con siRNA or HuD siRNA for 4 days, lysed, and then used for immunoprecipitation using NEP antibody. Immunocomplexes were further analyzed to detect phosphorylated and biotinylated NEP protein in immunoblot analyses after normalization for NEP protein level.

NEP protein cellular localization and enzymatic activity are highly dependent on its phosphorylation status [33], [34]. We determined whether the increased NEP expression levels induced by PKCε could leads to an increase in the activity of NEP by examining phosphorylation status and membrane localization. First we found that bryostatin treatment for 1 hr didn’t induced NEP protein expression. To test the phosphorylation status of NEP protein after PKCε activation, we treated cells with bryostatin for 1 hr, immunoprecipitated NEP from the lysates, and then performed immunoblot analyses using a phospho-Ser/Thr antibody. Bryostatin treatment increased NEP phosphorylation by approximately 2-fold, whereas pre-treatment with Ro 32-0432 almost completely blocked this effect (Fig. 4A). To investigate the membrane-localized NEP protein level, cells incubated with bryostatin for 1 hr were treated with biotin solution to label membrane-localized proteins. After immunoprecipitation using NEP antibody and normalization of NEP protein level, biotinylated NEP protein was detected by immunoblot analyses. Bryostatin treatment induced biotinylation of NEP protein by approximately 2.1-fold, indicating increased membrane localization after PKCε activation, an effect that was almost completely blocked in cells pre-treated with Ro 32-0432 (Fig. 4A).

To further examine the effect of PKCε on NEP activation, we suppressed PKCε expression with specific siRNA against PKCε and measured NEP phosphorylation status and biotinylated NEP protein levels by immunoblot analysis. NEP protein levels were normalized to account for the decreased NEP protein level in cells transduced with PKCε-specific siRNA. PKCε silencing dramatically reduced the levels of phospho-Ser/Thr and biotinylated NEP protein by ∼70% and 75%, respectively. Under PKCε silencing conditions, treatment with bryostatin did not have an effect on NEP phosphorylation or membrane localization (Fig. 4B). These data strongly suggest that activated PKCε phosphorylates NEP protein and enhances its translocation to the cell membrane.

We also tested whether HuD protein is involved in the phosphorylation status and membrane localization of NEP protein. In cells transfected with HuD-specific siRNA, we measured the phosphorylation status and biotinylated levels of NEP protein in immunoblot analyses after normalization of NEP protein level, to account for decreased NEP protein level in cells transduced with HuD-specific siRNA (Fig. 4C). We found no difference in phosphorylation status or biotinylated levels of NEP protein, indicating that HuD plays a role in NEP mRNA stability and protein expression.

### PKCε Activation Enhances NEP Activity

**Figure 5:**
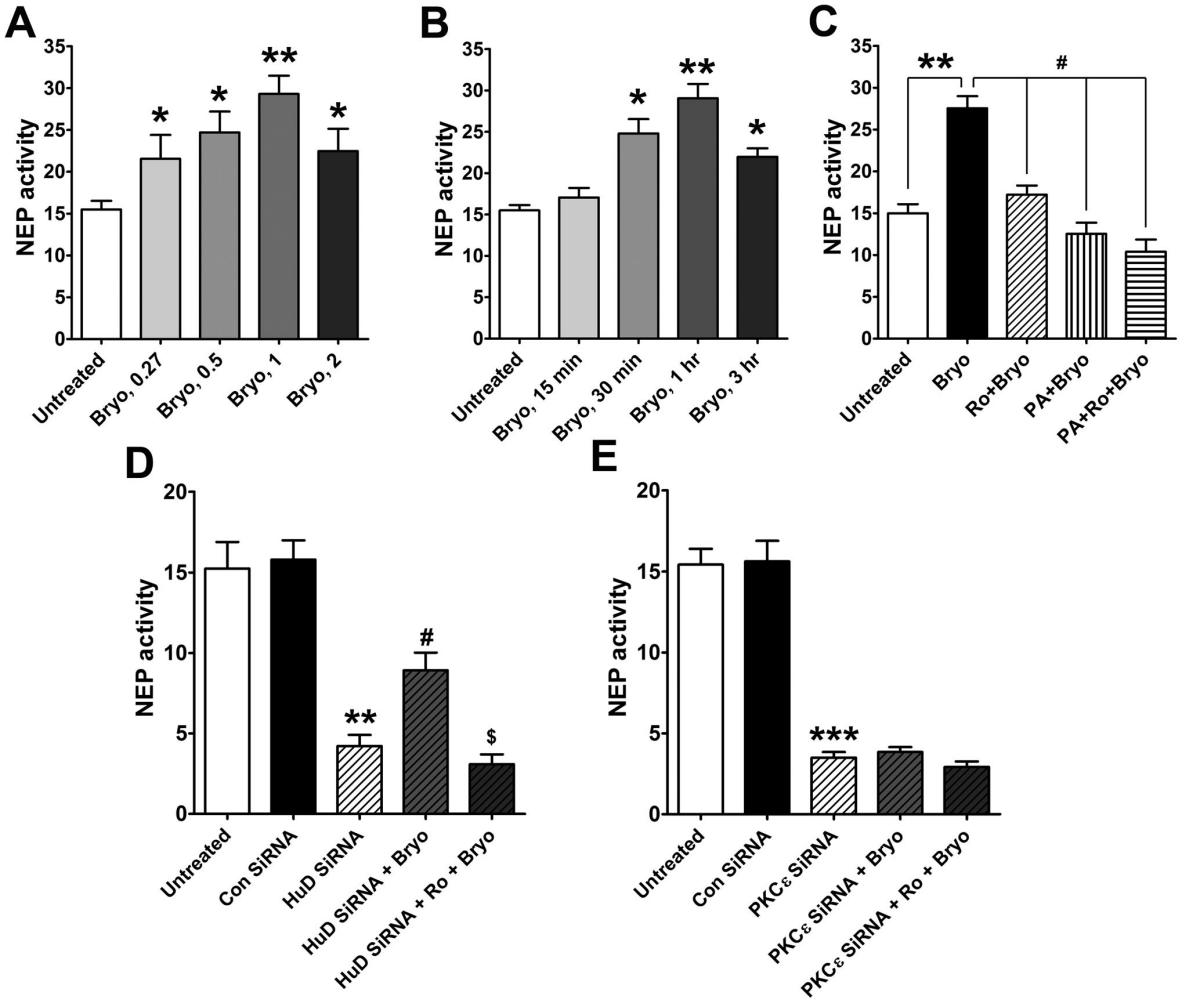
PKCε activation enhances NEP activity. **A**, Fluorometric measurement of NEP activity from cells in the absence (untreated) or presence of bryostatin (Bryo) at 0.27, 0.5, 1, or 2 nM for 1 hr (Mean ± SEM of three independent experiments, *P<0.05, **P<0.01, Bryo compared with untreated). **B**, NEP activity in cells in the absence (untreated) or presence of 1 nM Bryo for 15 min, 30 min, 1 hr, or 3 hr (Mean ± SEM of three independent experiments, *P<0.05, **P<0.01, Bryo compared with untreated). **C**, NEP activity in cells in the absence (untreated) or presence of 1 nM Bryo, pre-incubated with 2 µM Ro for 30 min, pre-incubated with phosphoramidon (PA, 10 µM, a specific NEP inhibitor) for 5 min, or pre-incubated with Ro+PA before Bryo treatment for 1 hr (Mean ± SEM of three independent experiments, **P<0.01, compared with untreated; ^#^P<0.01, compared with Bryo). **D**, NEP activity measurement in untreated cells or cells incubated with control siRNA (Con siRNA) or HuD siRNA without or with treatment of 1 nM Bryo or 2 µM Ro+Bryo for 1 hr (Mean ± SEM for three independent experiments, **P<0.01, compared with Con siRNA; ^#^P<0.05, compared with HuD siRNA; ^$^P<0.05, compared with HuD siRNA+Bryo). **E**, NEP activity measurement in untreated cells or cells incubated with Con siRNA or PKCε siRNA without or with treatment of 1 nM Bryo or 2 µM Ro+Bryo for 1 hr (Mean ± SEM for three independent experiments, ***P<0.001, compared with Con siRNA).

Using a fluorometric peptide substrate, we measured NEP activity change in human cells treated with different concentrations of bryostatin for 1 hr. At 0.27 nM, bryostatin treatment slightly increased NEP activity and 0.5 nM of bryostatin significantly increased NEP activity (Fig. 5A). Cells treated with 1 nM bryostatin showed highest NEP activity (approximately 2-fold), and NEP activity was slightly lower in cells treated with 2 nM bryostatin. A time course study in cells treated with bryostatin at 1 nM showed that bryostatin treatment significantly increased NEP activity at 1 hr, with a decrease in NEP activity by 3 hrs (Fig. 5B). To further investigate the role of PKCε in specific induction of NEP activity, cells were pre-treated with a PKCε inhibitor Ro 32-0432, a specific NEP inhibitor phosphoramidon, or both inhibitors prior to bryostatin treatment. PKCε inhibition with Ro 32-0432 pre-treatment significantly reduced bryostatin-induced NEP activation. Incubation with phosphoramidon completely inhibited bryostatin-induced NEP activity. Cells treated with both inhibitors showed a further decrease in NEP activity (Fig. 5C).

We further determined the roles of HuD and PKCε in NEP activation in cells transduced with HuD- or PKCε-specific siRNA compared to untreated cells. HuD silencing decreased NEP activity by ∼73%. PKCε activation with bryostatin treatment increased NEP activation by approximately 26%, compared with untreated cells under HuD silencing conditions; this effect was completed blocked by Ro 32-0432 pre-treatment (Fig. 5D). In addition, we found that PKCε silencing decreased NEP activity by ∼80%, and that there was no significant increase in NEP activity upon bryostatin treatment of cells transduced with PKCε-specific siRNA (Fig. 5E). These results strongly indicate that activated PKCε promotes NEP activation in SK-N-SH cells.

### Activated PKCε Protects SK-N-SH Cells against Aβ Peptide Accumulation and Toxicity through NEP Stabilization and Activation

**Figure 6:**
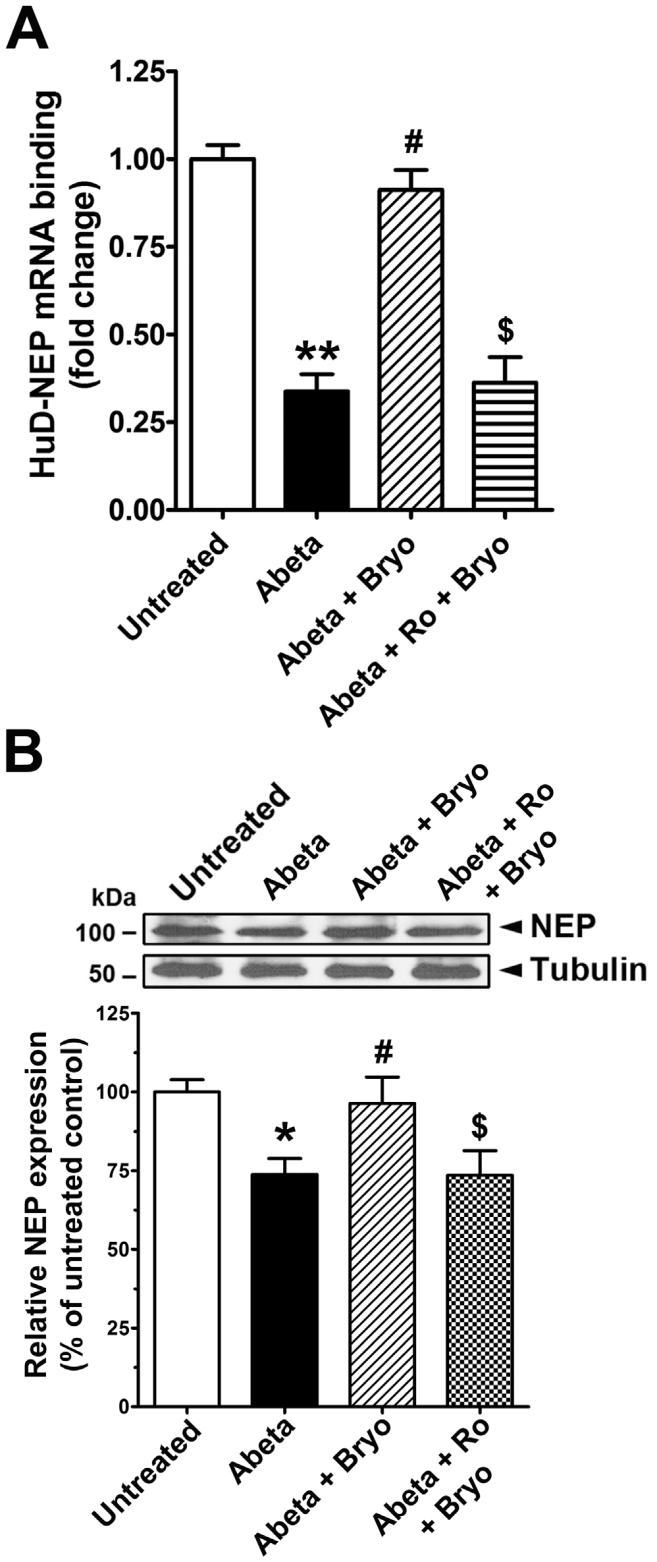
PKCε activation rescues oligomeric Aβ-mediated inhibition of HuD-NEP mRNA interaction and NEP protein expression. **A**, RIP analysis to detect NEP mRNA associated with HuD protein in untreated cells or cells treated with Aβ (Abeta, 1 µM), Aβ+bryostatin (Bryo, 0.5 nM) or pre-treated with Ro 32-0432 (Ro, 2 µM) for 30 min and then treated with Abeta+Bryo for 6 hr. Relative amounts of NEP mRNA bound to HuD were analyzed by real-time RT-qPCR (Mean ± SEM for three independent experiments, **P<0.01, compared with untreated; ^#^P<0.01, compared with Abeta; ^$^P<0.01, compared with Abeta+Bryo). **B**, Relative change in NEP protein expression was determined based on immunoblot analyses of untreated cells or cells treated with 1 µM Abeta or Abeta +0.5 nM Bryo, or pre-treated with 2 µM Ro for 1 hr prior to Abeta+Bryo for 24 hr (Mean ± SEM of the three independent experiments, *P<0.05, compared with untreated; ^#^P<0.05, compared with Abeta; ^$^P<0.05, compared with Abeta+Bryo).

We showed previously that Aβ inhibits PKCε and HuD activation and that bryostatin treatment can reverse the effects of Aβ on HuD and synaptic loss. We wanted to test whether oligomeric Aβ inhibits the HuD-NEP mRNA interaction and if activated PKCε can rescue the Aβ effect. Cells treated with oligomeric Aβ showed a dramatic decrease in HuD-NEP mRNA binding (Fig. 6A). Co-treatment with bryostatin rescued the decrease in the HuD-NEP mRNA interaction, an effect that was completely blocked by PKCε inhibition (Fig. 6A). Oligomeric Aβ-treated cells also showed a significant decrease in NEP protein levels, which was completely rescued by co-treatment with bryostatin (Fig. 6B). This effect was also completely blocked by PKCε inhibition.

**Figure 7:**
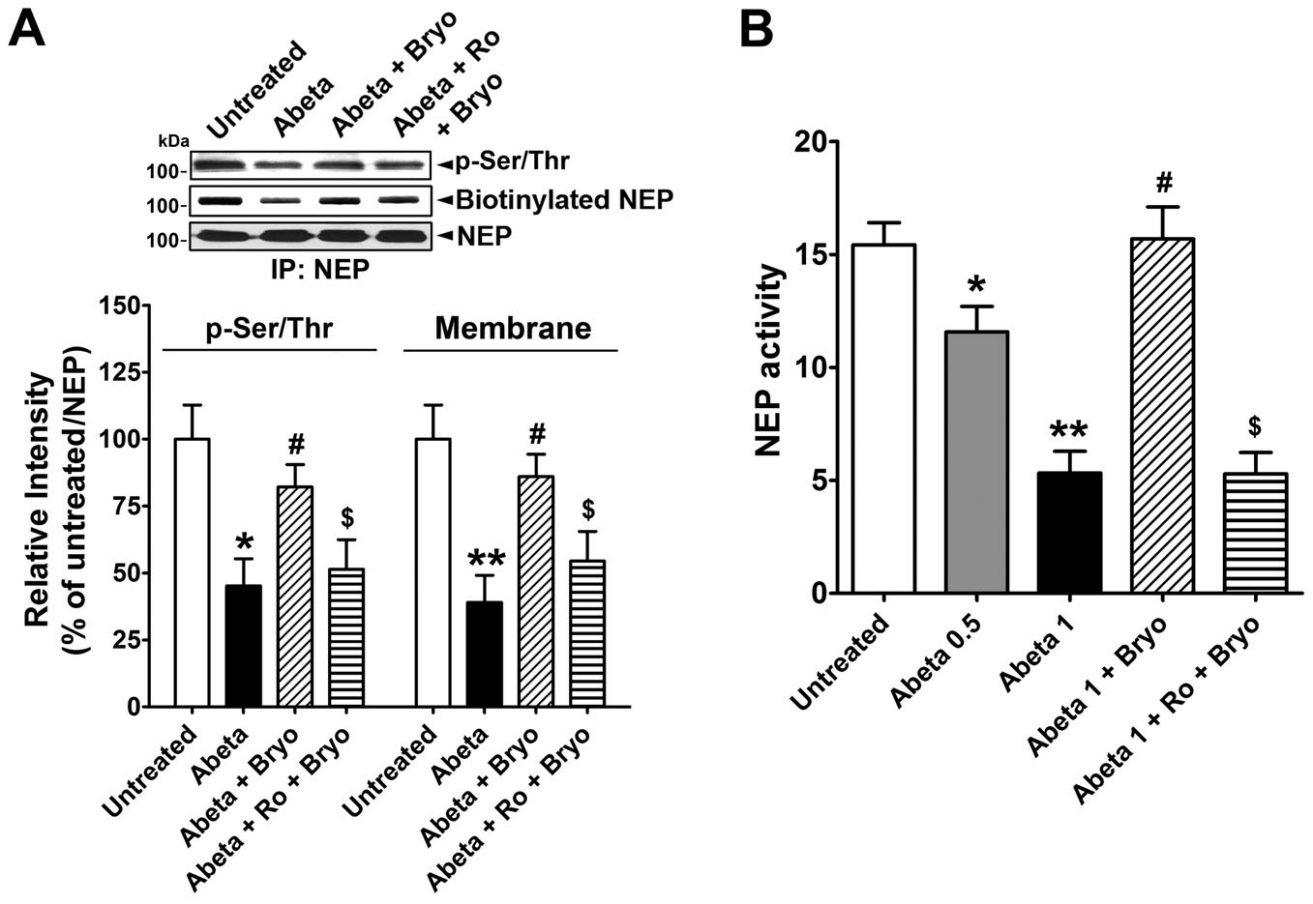
PKCε activation recovers NEP membrane localization inhibited by oligomeric Aβ peptides. **A**, Cells were untreated or treated with oligomeric Aβ (Abeta; 1 µM) or Abeta+bryostatin (Bryo, 1 nM), or pre-incubated with Ro 32-0432 (Ro, 2 µM) for 30 min before Abeta+Bryo treatment for 1 hr, and then used for biotin-labeling. Phosphorylated and biotinylated NEP proteins were detected by immunoblot from immunoprecipitated NEP protein and were compared (Mean ± SEM of the three independent experiments, *P<0.05, **P<0.01, compared with untreated; ^#^P<0.05, compared with Abeta; ^$^P<0.05, compared with Abeta+Bryo). **B**, NEP activity assay from untreated cells or cells treated with 0.5 or 1 µM Abeta, or Abeta +1 nM Bryo, or pre-incubated with 2 µM Ro for 30 min before Abeta+Bryo treatment for 1 hr (Mean ± SEM for three independent experiments, *P<0.05, **P<0.01, compared with untreated; ^#^P<0.01, compared with Abeta; ^$^P<0.01, compared with Abeta+Bryo).

We then tested whether Aβ exposure inhibits NEP phosphorylation, membrane localization, and activity, and whether bryostatin activation of PKCε could reverse these effects. Oligomeric Aβ-treated cells showed a significant decrease in phosphorylated and biotinylated NEP protein by ∼60% and 70%, respectively, indicating a decrease in cell membrane localization of NEP protein. Co-incubation with bryostatin effectively rescued oligomeric Aβ-inhibited phosphorylation and membrane localization of NEP protein by ∼40% and 50%, respectively, which were almost completely blocked by PKCε inhibition (Fig. 7A). We then tested NEP activity in cells treated with oligomeric Aβ (Fig. 7B). Treatment of cells with oligomeric Aβ at 0.5 or 1 µM reduced NEP activity by 25% or 70%, respectively, consistent with decreased phosphorylation status and membrane localization level of NEP protein. Co-treatment with bryostatin effectively recovered the Aβ-mediated decrease in NEP activity; this effect was completely blocked by PKCε inhibition with Ro 32-0432 pre-treatment.

**Figure 8:**
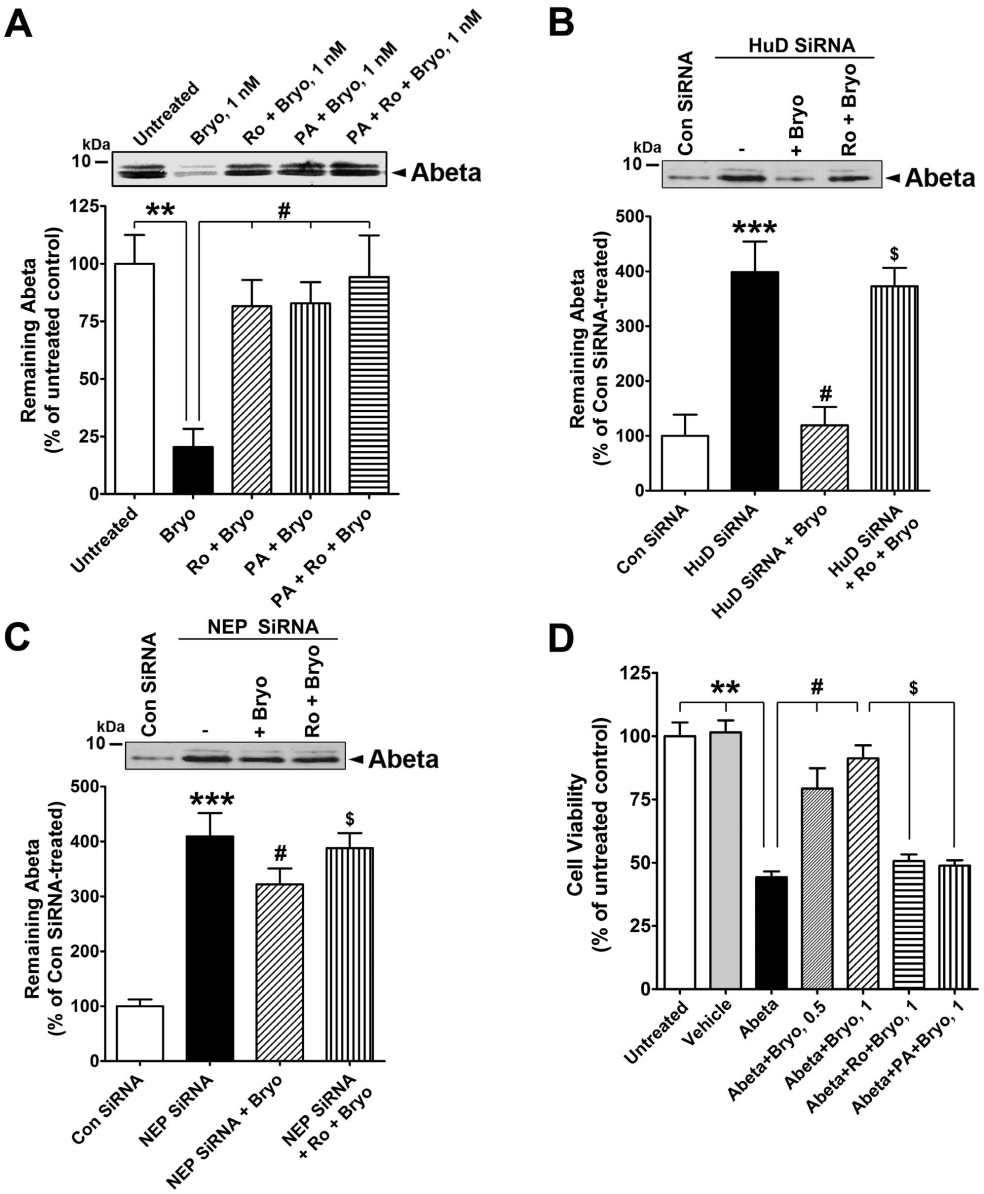
Activated PKCε stimulates NEP activity to protect SK-N-SH cells against Aβ neurotoxicity. **A**, Cells were untreated or treated with bryostatin (Bryo, 1 nM), phosphoramidon (PA, 10 µM)+Bryo, Ro 32-0432 (Ro, 2 µM)+Bryo, or PA+Ro+Bryo for 1 hr and incubated with monomeric Aβ 1–42 peptide (Abeta, 2.5 µg) for additional 4 hr. Aβ 1–42 peptide was precipitated from the reactions by 20% TCA and immunoblotted with anti-Aβ peptide antibody 6E10 (Mean ± SEM for three independent experiments, **P<0.01, compared with untreated; ^#^P<0.05, compared with Bryo). **B**, Aβ degradation assay in cells incubated with control siRNA (Con siRNA) or HuD siRNA without or with treatment of bryostatin (Bryo, 1 nM) or Ro 32-0432 (Ro, 2 µM)+Bryo for 1 hr (Mean ± SEM for three independent experiments, ***P<0.001, compared with Con siRNA; ^#^P<0.001, compared with HuD siRNA; ^$^P<0.001, compared with HuD siRNA+Bryo). **C**, Aβ degradation assay in cells incubated with Con siRNA or NEP siRNA without or with treatment of 1 nM Bryo or 2 µM Ro+Bryo for 1 hr (Mean ± SEM for three independent experiments, ***P<0.001, compared with Con siRNA; ^#^P<0.05, compared with NEP siRNA; ^$^P<0.05, compared with NEP siRNA+Bryo). **D**, Cells were incubated in absence (untreated) or presence of DMSO (vehicle) or oligomeric Aβ1–42 peptide (1 µM) for 24 hr. Cells were co-treated with Abeta +0.5 or 1 nM Bryo or pre-treated with 2 µM Ro for 1 hr or phosphoramidon (PA, 10 µM) for 10 min prior to co-treatment with Abeta+Bryo (1 nM). The viability of the cells after treatment was determined by cell viability assay and results are expressed as a percentage of viable cells compared with untreated control cells (Mean ± SEM of three independent experiments, **P<0.01, compared with untreated; ^#^P<0.05, compared with Abeta; ^$^P<0.01, compared with Abeta+Bryo).

To show that PKCε-induced NEP activity degrades Aβ, human neuroblastoma cells were treated with bryostatin with or without PKCε inhibitor or NEP inhibitor and then exposed to monomeric Aβ 1–42 peptides. Aβ peptide degradation assays showed that cells treated with bryostatin contained less Aβ peptide, indicating that activated PKCε increased NEP activity to effectively degrade the Aβ 1–42 peptide. In contrast, co-treatment with either Ro 32-0432 or phosphoramidon almost completely blocked the effect of bryostatin on Aβ degradation (Fig. 8A). To demonstrate whether the effect of bryostatin on increased Aβ degradation is dependent on HuD or NEP protein level, we examined Aβ peptide degradation assays in cells transduced with HuD- or NEP-specific siRNA. HuD silencing dramatically inhibited Aβ degradation. PKCε activation was able to enhance Aβ degradation, which was almost completely blocked by PKCε inhibition (Fig. 8B). NEP silencing also inhibited Aβ degradation. PKCε activation slightly increased Aβ degradation, which was completely blocked by PKCε inhibition (Fig. 8C).

To further demonstrate that the protective effect of activated PKCε against Aβ peptide toxicity in cells occurs through NEP activation, cells were incubated for 24 hr with culture media containing 2% FBS with or without oligomeric Aβ peptide (1 µM). Cells were co-treated with bryostatin and either Ro 32-0432 or phosphoramidon for 24 hr. At the end of the incubation period, cell viability was quantified. The viability of neuroblastoma cells was significantly decreased by oligomeric Aβ peptide; viability was increased approximately 30% by 0.5 nM bryostatin treatment and 35% by 1 nM bryostatin treatment. Ro 32-0432 and phosphoramidon almost completely blocked the protective effect of bryostatin, indicating that activated PKCε increases NEP activity to effectively reduce Aβ levels and protect SK-N-SH cells against Aβ peptide toxicity (Fig. 8D).

## Discussion

We found that PKCε-regulated HuD protein interacts with the Aβ-degrading enzyme NEP mRNA and increases its stability and expression, and that activated PKCε is critically important for NEP localization and activation, leading to decreased Aβ levels in cultured human neuroblastoma SK-N-SH cells. These findings are consistent with previous studies by our group, which found that treatment of a mouse model of AD with bryostatin resulted in a decrease in Aβ levels. Our study suggests that the observed effects of PKCε activation on Aβ degradation are mediated by HuD and the stabilization and activation of the Aβ-degrading enzyme NEP.

PKCε activation up-regulates HuD expression and stimulates its redistribution into the cytosol and dendrites of hippocampal neurons, where it subsequently controls post-transcriptional expression of target genes [18]. In addition, PKCε activator treatment of AD neuronal cells prevents degradation of HuD mRNA and HuD-associated mRNAs and restores decreased HuD protein (unpublished data). Our findings extend the current model of PKCε-mediated Aβ degradation to include the interaction of HuD with NEP mRNA. Bryostatin treatment reversed the inhibitory effects of Aβ on HuD-NEP mRNA binding and NEP protein expression.

NEP activity correlates to its localization to the cell surface, a process that is regulated by phosphorylation-dephosphorylation of the NEP intracellular domain [33], [34]. We found that activated PKCε signaling resulted in increased phosphorylation and cell membrane localization of NEP, although it remains unclear whether activated PKCε directly phosphorylates the NEP protein. Interestingly, casein kinase 2 has been identified as a phosphorylating kinase for NEP that specifically increases NEP activity [33]. Conversely, ERK-mediated phosphorylation decreases NEP Aβ-degrading activity [34], indicating that additional regulation factors might be involved, and future studies will be performed to determine whether PKCε plays a direct role in NEP protein regulation.

Our data further establish NEP as a major factor in the pathogenesis of AD. Aβ peptide in the brain plays a central role in the pathogenesis of AD [2], [4], and alterations in NEP activity likely affect the development and progression of the disease. In familial AD, mutations in APP and presenilins are linked to aberrant increases in the generation of neurotoxic Aβ peptides. However, in sporadic AD, which comprises over 90% of all AD cases, Aβ amyloidosis may be caused by a decline in Aβ degradation, Aβ clearance, or both. As a potential therapeutic approach, elevation of Aβ-degrading enzyme expression/activity could promote Aβ degradation and reduce the accumulation of both soluble and fibrillary Aβ in the AD brain [35], [36].

We previously showed that overexpressing PKCε in AD transgenic mice dramatically lowers Aβ and plaques in AD transgenic mice without affecting α-secretase or APP metabolism [37]. Our current finding–that bryostatin treatment dramatically reduced Aβ levels in monomeric Aβ-treated cells *in vitro*–agrees with our previous studies showing that in AD transgenic cell lines that overexpress Aβ genes, chronic administration of PKCε activators bryostatin, DCP-LA, or DHA-CP6 reduces Aβ secretion and Aβ accumulation, as well as increases ECE activation [18], [38]. In the Tg2576 AD mouse and aged rat models, chronic bryostatin treatment dramatically reduces the levels of Aβ, recovers the loss of neurotrophic activity and synapses, prevents neuronal apoptosis, inhibits tau phosphorylation by inhibition GSK-3β, and enhances synaptogenesis, leading to recovering cognitive deficits [18], [19]. Thus PKCε activators may further contribute to the treatment of AD, by supporting Aβ degradation via increased NEP activity. To further probe this, in vivo studies of HuD and NEP activity in various AD animal models are underway.

Taken together, our results suggest that PKCε triggers HuD-mediated binding and stabilization of NEP mRNA, which restores NEP expression and activity. In AD, Aβ proteins decrease HuD expression, and therefore NEP activity, which favors further Aβ accumulation and disease progression. PKCε activators may be potential therapies that can recover NEP protein levels and activity in the AD brain, resulting in a reduction of toxic Aβ levels, and restoration of synaptogenesis and cognitive function.
